# Isolation and Analysis of Phospholipids in Dairy Foods

**DOI:** 10.1155/2016/9827369

**Published:** 2016-08-17

**Authors:** Lígia Pimentel, Ana Gomes, Manuela Pintado, Luis Miguel Rodríguez-Alcalá

**Affiliations:** ^1^Centro de Biotecnologia e Química Fina (CBQF), Laboratório Associado, Escola Superior de Biotecnologia, Universidade Católica Portuguesa, Rua Arquiteto Lobão Vital, Apartado 2511, 4202-401 Porto, Portugal; ^2^Centro de Investigación en Recursos Naturales y Sustentabilidad (CIRENYS), Universidad Bernardo O'Higgins, Fábrica N° 1990, Segundo Piso, Santiago, Chile

## Abstract

The lipid fraction of milk is one of the most complex matrixes in foodstuffs due to the presence of a high number of moieties with different physical and chemical properties. Glycerolipids include glycerol and two fatty acids esterified in positions sn-1 and sn-2 with higher concentration of unsaturated fatty acids than in the triglyceride fraction of milk. Sphingolipids consist of a sphingoid base linked to a fatty acid across an amide bond. Their amphiphilic nature makes them suitable to be added into a variety of foods and recent investigations show that phospholipids, mainly phosphatidylserine and sphingomyelin, can exert antimicrobial, antiviral, and anticancer activities as well as positive effects in Alzheimer's disease, stress, and memory decline. Polar lipids can be found as natural constituents in the membranes of all living organisms with soybean and eggs as the principal industrial sources, yet they have low contents in phosphatidylserine and sphingomyelin. Animal products are rich sources of these compounds but since there are legal restrictions to avoid transmission of prions, milk and dairy products are gaining interest as alternative sources. This review summarizes the analysis of polar lipids in dairy products including sample preparation (extraction and fractionation/isolation) and analysis by GC or HPLC and the latest research works using ELSD, CAD, and MS detectors.

## 1. Introduction

The lipid fraction of milk is one of the most complex matrixes in foodstuffs since it is composed of tri- (TG), di- (DG), and monoglycerides (MG), glycolipids, cholesterol (CH), cholesterol esters (CHE), free fatty acids (FFA), phospholipids (PL), and sphingolipids (SPL) [1]. Such variety in the number and chemical nature of the compounds represents itself as a challenge when obtaining a detailed composition, requiring previous steps of isolation, purification, and/or fractionation of the target compounds eventually coupled to derivatization reactions [2–4] to perform analysis using GC or HPLC techniques. Furthermore, there is a renewed interest in the milk lipids since some investigations have demonstrated that certain compounds can exert biological effects as protective capabilities of MG against gastrointestinal infections [5, 6], anticancer properties of butyric, rumenic, odd chain fatty acids and ether lipids [7, 8]. Moreover, medium chain triglycerides (MCTG) can be used as an energy source in parenteral nutrition, pancreatic or bile secretion insufficiency as they are directly hydrolyzed by gastric lipase and absorbed without the need for reesterification [9–11].

In milk TG, which comprise 98% of total lipids, are enveloped to prevent its enzymatic degradation by lipases, inside the milk fat globule membrane (MFGM) which is composed of lipids (40%), proteins (60%), and CH from epithelial cells of the mammary gland, including significant amounts of PL [12].

Biological activities have been also described for PL as antioxidant [13, 14], antimicrobial, and antiviral properties [15], protective effect against gastric ulcers [16], important roles as active agents in colon cancer, reducing the risk of cardiovascular diseases [17], Alzheimer's, stress, and memory decline associated with phosphatidylserine [18], or implication in other mental disorders as depression [19, 20]. Most of them may be related to the presence of PUFAs in its sn-2 position [21, 22]. Recently, Reis et al. [23] reported that female mice fed a high-fat diet supplemented with bovine SPL showed a tendency for lower TG synthesis in the liver, but not in adipose tissue, while PL or PL + SPL reduced* de novo* hepatic fatty acid biosynthesis, therefore showing the potential of milk polar lipids as a functional ingredient to ameliorate the impact of a diet rich in fat.

On the other hand, some research studies conducted using the MFGM have showed its potential as nutraceutical or functional ingredient: piglets fed with a formula composed of polydextrose (1.2 g/100 g diet), galactooligosaccharides (3.5 g/100 g diet), bovine lactoferrin (0.3 g/100 g diet), and MFGM (2.5 g/100 g diet) for 30 days showed increased weight gain and gut maturation, modulated colonic and faecal microbial composition, and reduced proportions of opportunistic pathogens [24]. These results are not unexpected as previous authors found that MFGM from raw or heat-treated milk inhibited the expression of the Shiga toxin gene in* E. coli* O157:H7 [25] while in humans orally challenged with live, attenuated diarrheagenic* E. coli* the intake of a milk protein concentrate rich in MFGM improved the in vivo resistance to this bacteria [26].

Phospholipids and SPL compounds are also interesting for the food industry as emulsifiers as their amphiphilic nature makes them suitable to be added into a great variety of foods. They can be found as natural constituents in the membranes of all living organisms. Although soybean is the greatest source of PL, with a commercial production of 200 tons/year, and egg lecithin represents 300 tons/year, both have very low concentrations of glycerophosphatidylserine (GPS) and sphingomyelin (SM) [27]. Animal sources as brain and bone marrow are of interest since they have high concentrations of SM but their use is legally restricted to avoid transmission of prions related to diseases as bovine spongiform encephalitis, scrapie, and Creutzfeldt-Jacob disease [28].

Independently of the abovementioned advantages supporting dairy products as an interesting source of polar lipids for the elaboration of new products it must not be forgotten that processing can affect the composition of dairy lipids [29, 30]. Moreover, some recent studies have found that pasteurization of bovine milk resulted in lower concentrations of myristic acid (C14:0), palmitic acid (C16:0), oleic acid (C18:1 c9), and EPA (C20:5 n3) [31]. These authors also reported that homogenization led to a loss of glycerophosphatidylcholine (GPC) and SM in the outer layer and resulted in a higher proportion of glycerophosphatidylethanolamine (GPE) and GPS. Previously, studies conducted to investigate the effects of pasteurization, evaporation, and spray-drying on buttermilk reported that all these steps resulted in lower PL content related to GPE, GPS, and glycerophosphatidylinositol (GPI) [32].

The biological and industrial importance of dairy PL and SPL as bioactive/functional ingredients and the studies showing the changes due to processing thereof highlight the importance of the analysis of these compounds.

## 2. Contents and Distribution of Polar Lipids in Dairy Products

From the current bibliography, Rombaut et al. [33–35] carried out the most extensive analysis in dairy products reporting the content and distribution of PL in raw, UHT, pasteurized and sterilized milks, cheeses, cream, butter, buttermilk, and butterserum. However, these data have been further updated/completed by the works carried out on small ruminants' milk and some of its products by Yao et al. [36], Castro-Gómez et al. [37], Zancada et al. [38], Contarini and Povolo [39], Rodríguez-Alcalá and Fontecha [40] (this latter also focused on powder buttermilk), and Park [41]. Moreover Barry et al. [42] reported the PL composition of butter, butterserum, cream, butteroil, and whole and skimmed milk while Fong et al. [43] analysed infant formulas and Ferreiro et al. [44] described the evolution of PL concentration during the production of quark cheese. Such information is accordingly summarized in Table 1 (PL content in dairy products) and Table 2 (PL distribution in dairy products). As it can be observed, total content can vary from 278.8 mg PL/100 g product in Ricotta cheese to 1.12 mg/100 g product in buttermilk being 29.4–40.0 mg/100 g product in raw cows' milk. However, high PL dairy sources are low-fat products such as skimmed milk (11.1–19 g PL/100 g fat), buttermilk (up to 33 g PL/100 g fat), and butterserum (14.8–48.4 g PL/100 g fat). The milk phospholipids in cows' milk (Table 2) are GPE (23.4–46.7% of total PL), GPC (19.1–33.2%), SM (17.8–29.2%), GPS (2–9.07%), and GPI (3.98–8.97%).

In small ruminants' milk the amount is 27.6 mg/100 g in goats' and 29.8 mg/100 g product for ewes'. Regarding the compounds distribution (Table 2), for ewes' it is 26.1–40.0 g GPE/100 g PL, 26.4–27.2 GPC/100 g PL; 4.96–10.7 GPS/100 g PL; 4.16–6.40 GPI/100 g PL; and 22.6–29.7 SM/100 g PL while in goats' milk it has been reported as 19.9–41.4 g GPE/100 g PL; 27.2–31.9 g GPC/100 g PL; 3.2–14 g GPS/100 g PL; 4.00–9.37 g GPI/100 g PL; and 16.1–29.2 g SM/100 g PL. Furthermore, Zancada et al. [38] also reported the fatty acid (FA) composition of PL in these milks (Table 3), with palmitic (34% in GPC, 28.8% in GPS, 25.5% in GPI, 12% in GPE, and 25% in SM for ewes' milk; 40% in GPC, 13.3% in GPS + GPI, 21.2% in GPE, and 20.7% in SM for goats' milk), stearic (18% in GPC, 33.9% in GPS, 28.6% in GPI, 17.5% in GPE, and 6.3% in SM for ewes' milk; 8% in GPC, 47.9% in GPS + GPI, 12.4% in GPE, and 8% in SM for goats' milk), and oleic acid (32.3% in GPC, 16.7% in GPS, 25.2% in GPI, 53.6% in GPE, and 0.6% in SM for ewes' milk; 34.4% in GPC, 29.7% in GPS + GPI, 50.6% in GPE, and 0.9% in SM for goats' milk) as the main compounds. On the other hand, for cows' milk these FAs are also the most abundant in the composition of phospholipids: palmitic acid was 35.5% in GPE, 34.7% in GPC, 27.7% in GPS + GPI, and 18.9% in SM while stearic was 16.9% in GPE, 9.5% in GPC, 25.7% in GPS + GPI, and 4.3% in SM and oleic acid, 23.5% in GPE, 26.7% in GPC, 24.9% in GPS + GPI, and 0.5% in SM [45]. However, according to these latter authors, linoleic acid also showed important concentrations (6.6% in GPE, 15% in GPC, 7.2% in GPS + GPI, and 0.4% in SM) for some products.

It is well established that FA composition of milk is affected by animal (breed, state of lactation, and health), environmental (season), and feeding factors (this latter, largely used to increase the unsaturated FA composition while decreasing the saturated content in milkfat) [1]. However, these effects are not solely limited to the FA composition, as some other investigations have reported changes in the dairy PL associated to such factors. Bitman and Wood [46] studied the lipid composition of milk from Holstein cows postpartum (from day 3 to 180). According to the reported results, PL content increased from day 3 to 42 (0.72–1.11 g/100 g fat) to finally decrease (0.56 g/100 g fat at day 180). This pattern was similar for the distribution of GPE, GPC, and SM while GPI decreased along the assayed period. On the contrary, GPS amount increased from day 3 to 7, remaining constant after that.

Further research works compared the PL composition from cows in two different locations in Spain: although the total concentration was similar between the two milk batches, differences were found concerning the PL distribution of GPE (45.44% versus 33.94%), GPI (2.51% versus 3.65%), and SM (14.70% versus 24%) [47].

Other studies aimed to investigate the effect of feeding a maize silage versus the same diet supplemented with linseed on the MFGM phospholipids obtained from the milk of Holstein and Montbeliarde cows [48]. Addition of linseed resulted in a content of 2.98 mg PL/g lipid (and increment of all the PL compounds) while the amount in the MFGM from animals fed maize silage was 2.53 mg PL/g lipid. These results seem to be recently confirmed as cream obtained during the early phase of the Parmigiano-Reggiano cheese-making process using milk from Holstein cows fed with linseed had 3.84 mg PL/g fat (3.48 mg PL/g fat in the control group) [49]. However, regarding the PL distribution, these authors only found increments for GPE, GPC, and SM.

In further studies when cows were fed pasture during spring and corn silage in winter, the PL content in the MFGM from the collected milks was of 3.5 mg/g lipid versus 2.5 mg/g lipid, respectively, resulting also in lower concentrations for all the components in this fraction [50].

## 3. Extraction and Purification Procedures

### 3.1. Isolation Procedures

The first step in any analysis is the isolation of the target compounds. Regarding milk fat, neutral lipids can be easily removed from milk using hexane, chloroform, or other similar solvents while alcohols (mainly methanol) are used for polar lipids [51]. Several works have pointed out the importance of the extraction procedure. An incomplete recovery of polar lipids using the Rose-Gottlieb protocol (ammonia/ethanol and diethyl ether/petroleum) proposed as the standard method for milk fat isolation has been reported elsewhere [52]: GPI and/or GPS are missed because acidic conditions increase their solubility in water [42, 53]. One of the most widely used alternatives is that proposed by Folch et al. [54] using 20 volumes of 2 : 1 chloroform/methanol (CM). However, Bligh and Dyer [55] later reported a variation of that method using 4 volumes of 1 : 1 CM and describing the precise proportion of water (including that from the sample) in the mixture to assure the highest isolation yield. In 2001 both methods were compared to study their isolation efficiency and the results showed that in ≤2% fat content samples there was a good correlation between the two methods but at higher concentrations Folch achieved better isolation yields [56].

One of the problems of the utilization of chloroform is its toxicity; thus, Hara and Radin [57] developed a process applying 18 volumes of 3 : 2 hexane/isopropanol (HIP) while Cequier-Sánchez et al. [58] demonstrated that dichloromethane can be assayed without affecting the composition of the sample. In further research works, chloroform was substituted by methyl tert-butyl ether (MTBE) yielding also good results [59]. This alteration represents a very interesting approach as lipids are in the higher layer due to the lower density of MTBE (740 kg/m^3^) compared with methanol (792 kg/m^3^).

Some approaches to the development of faster methods (10 minutes) have been focused on the utilization of pressurized liquid extraction (PLE) for the isolation of milk fat from goats', ewes', and cows' milk with a dichloromethane/methanol solution (2 : 1 v/v) as solvent mixture at 10.3 MPa and 60°C [37]. These authors did not find differences between the molecular species composition of TG and the total fatty acid profile of the assayed milks by PLE or Folch's method.

Other issues concerning the use of CM systems are the fact that they are biphasic extraction procedures and the methanol-water phase retains up to 31% of the lipids, mainly the acidic polar moieties [60]. This author proposed the utilization of single CM mixtures (without addition of water) and when contrasting single CM versus biphasic, HIP, and utilization of n-butanol in the isolation of lipids, only the CM single-phase method resulted in a complete recovery [61]. However, a recent study comparing all these methods (but not single CM), concluded that solvent composition has a small effect on the extraction of predominant lipid classes but isolation of less abundant lipids was greatly influenced by the solvent system used [62].

### 3.2. Fractionation Methods

After isolation of total lipids, fractionation steps can be performed prior to analysis by open column chromatography (disadvantageous because of the need for large amounts of sample and solvent while columns have to be manually packed), TLC (low load sample capacity, compounds have to be scratched off from plate and reextracted with solvents), or SPE columns. This latter method is commonly assayed due to the availability of a wide variety of commercial products, therefore assuring the stability of the stationary phases (bulk silica may lose activity when stored) has also a high reproducibility since columns present a homogeneous density and finally fractions are recovered directly in solvents while in TLC they have to be scrapped [63].

Nevertheless, TLC performed on precoated silica gel 60 plates developed with light petroleum/diethyl ether/acetic acid (90 : 10 : 1) followed by acetone was used to separate milk lipids into TG, MG, CH, CHE, FFA, and PL [64]. Scratching of the PL band and using a second silica plate with ethyl acetate/2-propanol/water (50 : 35 : 15) and chloroform/methanol/water (75 : 25 : 4) allowed the separation of PL into GPE, GPI, lysophosphatidylethanolamine (LPE), GPS, GPC, and SM.

The separation of different PL classes can be improved by using two-dimensional TLC (2D-TLC) as MacKenzie et al. [65] showed in the analysis of commercial fresh cream, fresh butterserum, and buttermilk protein concentrates. Thus, in a first development chloroform/methanol/28% aq. NH_3_/benzene (65 : 30 : 6 : 10 v/v) is applied while a second step involving chloroform/ethylacetate/acetone/2-propanol/ethanol/methanol/water/acetic  acid (30 : 6 : 6 : 6 : 16 : 28 : 6 : 2 v/v) achieves the separation of the acidic polar lipids (GPI, GPS, Lac-PE). Therefore, under these conditions dihydrosphingomyelin, ethylenediaminetetraacetic acid, Lac-PE (Amadori product of lactose and GPE), lysophosphatidylcholine, LPE, GPC, GPE, GPI, GPS, SM, and phosphatidic acid (PA) can be isolated. Wang et al. [66] previously reported the utilization of chloroform/methanol/ammonia (65 : 35 : 8 v/v) followed by chloroform/methanol/acetone/acetic acid/water (60 : 15 : 20 : 10 : 5 v/v) for the separation of GPE, GPI, GPS, GPC, and SM in human milk.

SPE avoids some of the problems of TLC: lipids are eluted dissolved in organics and cartridges can be cleaned and reutilized. The method proposed by Kaluzny et al. [67] assayed aminopropyl disposable columns (500 mg) and chloroform-2-propanol to elute TG, DG, MG, CH, and CHE (nonpolar lipids, neutral lipids) while FFAs were isolated with 2% acetic acid in diethyl ether and the whole PL with methanol. Under these conditions, only one single cartridge was needed. This method was further slightly modified (2 g aminopropyl columns versus 500 mg in the original method) to report the lipid composition of anhydrous butterfat and dry whey products, while the data was compared with those from TLC showing a good correlation [68].

In 2005, the utilization of silica gel bonded columns (1 g) was described for the separation of neutral lipids (8 : 2 hexane/diethyl-ether followed of 1 : 1 hexane/diethyl-ether) and PL (methanol followed by 3 : 5 : 2 chloroform/methanol/water) [53]. This latter method has been extensively used with dairy samples. It is indeed very similar to that previously developed by Bitman et al. [69] to analyze the phospholipid composition of human milk from mothers of term and preterm infants during lactation. According to these authors, neutral lipids are isolated using 1 : 1 hexane/ethyl ether and polar lipids with 3 : 5 : 2 chloroform/methanol/water, also in silica gel cartridges.

Further research works have studied other solvent combinations as cyclohexane/ethyl acetate followed by ethyl acetate/methanol, methanol, and finally methanol/H_2_O in silica gel columns to develop a method for the routine analysis of branched chain fatty acids in both neutral and polar lipids from cheese and fish samples [70].

Additional studies conducted on buttermilk isolating total lipids according to the Mojonnier method and PL by the procedures of Bitman et al., Avalli et al., and Vaghela et al. found that yields were 33.6, 29.6, and 20.7 nmol/mg lipid weight, respectively [71]. Moreover, the results from this study showed that assaying Bitman et al. method resulted in higher concentrations of GPC (47%, 9.7%, and 14.2%, resp.) and SM (22.6%, 0.6%, and 2%) while Avalli et al. and Vaghela et al. procedures were more suitable for GPE (9.6%, 34.5%, and 36.3%) and GPI (10%, 38.3%, and 35.9%).

Some researchers use clean-up procedures in order to eliminate proteins and PL from their samples, as they can lead to ion suppression in analysis involving mass spectrometry [72]. Thus, in the last years, SPE columns using zirconia-coated silica have been developed which, in combination with a precipitation solution, retains both protein and PL. It could then be thought that these zirconia SPE columns may result in an easier way to isolate PL. Nevertheless, studies conducted on butter fat spiked with a PL mix (GPE, GPI, GPS, GPC, PA, SM, and phospahtidylglycerol (PG) to a spiking concentration of 1 *μ*g/mL of each analyte) and comparing recoveries using C8, silica, and zirconia SPE columns found that using these latter, it was possible to recuperate 94.6% of total PG spiked and 92.9% of GPE [73]. Furthermore, the results for GPS, GPC (72.5% and 86.8%), PA, and SM (36.3% and 25%) were unsatisfactory and GPI was not recovered at all. These authors also reported that when using C8 columns it was not possible to retrieve PG and for the rest of compounds, recovery values were below 80%, even reaching 49.6% for GPS or 59.8% for SM. The best results were obtained assaying silica columns as values were above 90% for PG, GPE, GPI, GPS, GPC, and SM while 89.6% for PA.

Finally, as in the 2D-TLC, the separation of PLS into classes would be desirable. Thus recently, the conditions to separate PLS from complex matrices have been elsewhere described, after elimination of nonpolar lipids, as fraction 1 (GPC, LPC, GPE, and SM later divided as GPE and GPC + LPC + SM), fraction 2 (PG and 58.9% cardiolipin, CL), and fraction 3 (PA, GPI, GPS, and 38.9% CL) [74].

## 4. Quantification by Spectrophotometric and NMR Techniques and Structure Determination by MALDI-TOF

Despite the utilization of TLC and SPE methods for the isolation and fractionation of PL and their determination by weight, other authors have proposed some alternatives on the basis of the determination of phosphorus content. Thus, Stewart [75] developed a method in which, after isolation of lipids using CM, phospholipids were complexed with ammonium ferrothiocyanate and the optical density measured at 488 nm. According to the author, with this procedure it is possible to measure PL concentrations of 0.01–0.1 mg.

On the other hand, the total content of phospholipids (after elimination of neutral lipids in the samples by SPE) from raw and skim milk, cream, butter, buttermilk, and butterserum was quantified using the reduction of phosphomolybdate to blue colours at 830 nm, according to the method of Britten et al. [76]. Other strategies have isolated ewes' and goats' milk PL by TLC and determined the phosphorus content after hydrolysis of the samples with HCLO_4_, multiplying the results by a conversion factor of 25 [77].

In spite of all the above mentioned methods that required the isolation of total lipids followed by purification of PL by TLC or SPE, MacKenzie et al. [65] proposed a procedure in which dairy lipids were treated with a detergent solution and added with phosphonomethylglycine as internal standard to measure (mol%) PA, GPC, GPI, GPE, GPS, SM, LPC, LPE, DHSM, and DHSM + SM by ^31^P NMR in cream and other dairy products. In further studies, other authors assayed this kind of spectroscopy after dissolving fats from cows', mares', camels', and human milk of a mixture of deuterated chloroform/methanol/cesium cyclohexanediamine tetraacetic acid (CsCDTA) 5 mM in H_2_O (100 : 40 : 20, v/v/v) for the determination of plasmalogens, GPE, GPI, GPS, GPC, PA, SM, CL, LPA, LPE, LPC, and alkyl-acyl phosphatidylcholine [78].

It should be noted that milk and dairy products are rich in organic and inorganic compounds which can be coextracted with the lipid fraction leading to overestimation in the spectrophotometric and colorimetric analysis; besides nonphosphorous polar lipids such as glycosphingolipids are not measured.

On the other hand, some works aimed at the determination of the PL structure through MALDI-TOF/MS analysis. One of the first research works was that of Calvano et al. [79] performed on milk, chocolate milk, and butter. Prior to analysis, lipids from these samples were dissolved in dihydroxybenzoic acid (DHB, 10 mg/mL in 0.1% TFA) and loaded into TiO_2_ microcolumns to separate polar from neutral lipids. The eluted fraction containing the polar lipids was directly placed in the target plate. The ions from the DHB matrix were generated using a nitrogen laser at 337 nm (20 kV; 300 laser shots) while the mass spectra were acquired with a low-mass gate of 400 Da. According to these authors, although the identification of high molecular weight TG was possible, low molecular weight species identification was particularly difficult as an ion at a particular mass could correspond to losses of RCOO^−^Na^+^ or RCOO^−^K^+^ molecules from TG or to shorter TAGs species containing butyric acid (4 : 0). Concerning PLS, surprisingly only species of GPC, SM, and GPE were reported. In subsequent research works, to increase ionization efficiencies and facilitate lipids structural analysis, solutions of sodium or lithium chloride salts were mixed with DHB matrix solution and assayed in a MALDI-Q-TOF MS [80]. It was concluded from the obtained data that the alkaline adducts allowed a more precise determination of the polar head groups of different glycerophospholipid classes while providing information about the position (sn-1 and sn-2) of the fatty acid residue on the glycerol backbone. Nevertheless, in the assayed conditions, sodiated GPC precursors ions showed a poor fragmentation while in the case of GPI, the [M + H]^+^ adduct was never observable in the spectra (explaining thus their previous above mentioned results).

Recently, PL from raw and powder milk were purified using 500 mg silica gel cartridges, injected into HPLC system, collected one by one as eluted from the column, then dissolved in methanol, and identified by MALDI-TOF/TOF MS, acquiring all the spectra in reflector positive and negative mode within* m/z* range of 200–1600 [81]. Under these conditions, it was found that the molecular species according to their carbon atom number (CN) for GPC were comprised from 34 to 52, CN39 to CN44 for GPE, CN40 to CN42 for GPS, CN35 to CN45 in SM, CN43 to CN49 for GPI (detected in negative mode), and CN22 to CN28 for LPC. These authors were able to detect the FA esterified in the sn-1 and sn-2 positions of these PL, reporting that as expected they were mostly unsaturated except for SM where these compounds were predominantly saturated (C16, 18, C20, C12:1, and C22:3).

In 2015, shotgun lipidomics was tested for the profiling and quantification of lipids in human, cow, and a phospholipid-enriched milk formula [82]. After addition of internal standards and isolation, lipid extracts were subjected to MS/MS operating in positive and negative ion mode and programmed to perform sequential experiments in 1 amu steps across the precursor* m/z* range of 400–1000 and recording time-of-flight (TOF) MS/MS spectra with* m/z* range of 150–1000. Thus, it was possible to quantify the mol% of TG, GPC, GPE, SM, GPS, and GPI as well as the molecular species composition of TG and glycerophospholipids.

## 5. GC Analysis

In the profiling of dairy products, the analysis of TG has been successfully accomplished by using apolar columns attached to GC-FID [47, 83]. GC was also assayed for the analyses of DG and MG after TLC or SPE fractionation to eliminate neutral lipids and further conversion into their trimethylsilyl (TMS) derivatives to increase their volatility [84, 85]. As the structure of glycerophospholipids is highly similar to that of DG (glycerol, two fatty acids at sn-1 and sn-2 positions while sn-3 being esterified to phosphoric acid presenting up to seven possible residues; hydrogen, serine, ethanolamine, choline, inositol, glycerol, or phosphatidylglycerol) it could be thought that they may be also analysed by GC. However, PL are highly prone to deterioration by temperature [32, 86]. Therefore, to become GC compatible, these compounds have usually been hydrolyzed by phospholipase C and derivatized with tert-butyl-dimethylsilyl (TBDMS), trimethylsilyl (TMS), and pentafluorobenzoyl (PFB) [87]. On the other hand, for the analysis of sphingolipids by GC, some authors have proposed methods involving the release of the target compound using barium hydroxide, their oxidation into aldehydes by metaperiodate salts, and finally quantification of these aldehydes by GC-MS [88]. However, all these derivatization reactions add extra steps to the preparation of the samples that together with those of isolation/fractionation result in costly and cumbersome procedures.

## 6. HPLC Analysis

The analysis of polar lipids by HPLC allows bypassing the problems of the above mentioned techniques: lipids can be separated according to polarity without previous purification, fractionation, or derivatization. Furthermore, it does not require high instrument investments and highly experienced personnel as with ^31^P NMR or MALDi-TOF MS techniques. One of the most successfully assayed detectors in these determinations is the evaporative light scattering detector (ESLD). It has the particularity of being compatible with gradient elution and presenting insensibility to temperature variation [89]: The mobile phase and analyte are sprayed using a pneumatic nebulizer and the droplets directed toward a heating chamber, where solvent evaporates and nonvolatile compounds droplets scatter a light beam. Such detectors are mass sensitive (signal is consistent with power model equations), universal, and compatible with most of the solvents.

Christie [90] published one of the first reports using an ELSD (40°C, 27 psi) coupled to a 3 *μ* silica column, assaying a gradient of isooctane/tetrahydrofuran (99 : 1 v/v), isopropanol/chloroform (4 : 1 v/v), and isopropanol/water (1 : 1 v/v) for the separation of CH, CE, TG, MG, DG, FA, CER (cerebrosides), DPG (diphosphatidylglycerol), PG, GPE, GPI, GPC, SM, PMME (phosphatidylmonomethylethanolamine), LPE, PDME (phosphatidyldimethylethanolamine), GPS, and LPC in biological samples.

An alternative to ELSD is the charged aerosol detector (CAD) where the stream leaving the nebulizer is met by a secondary stream positively charged as a result of having passed a high-voltage, platinum corona wire. Thus, the charge transfers diffusionally to the opposing stream of analyte particles. Ramos et al. [91] compared the PL analysis from* Leishmania donovani* parasites using ELSD versus CAD attached to a 5 *μ*m PVA-Sil column, concluding that response from CAD followed a linear model while sensitivity was up to 10-fold higher for CAD than with ELSD.

As previously discussed in this current text, one of the main difficulties in the analysis of dairy PL is their low amounts, therefore requiring concentration and removal of TG prior to the analysis. Commonly, these preparative steps can be performed by TLC and/or SPE but also by using a silica guard column (PVA-Sil) in HPLC system [93]. Thus, milk fat is loaded into the guard column and neutral lipids removed using dichloromethane/2,2,4-trimethylpentane. Afterward, loop valves are switched to send the PL into a PVA-Sil analytical column. These authors reported that some stabilizers (mainly amylene) added to the dichloromethane resulted in the elution of an unknown peak close to the PL chromatographic region but treating this solvent with water eliminated this peak. Also in this work, to increase the response of the ELSD detector triethylamine (TEA) and formic acid (7.2 mM each) were added to the mobile phases but this resulted in deterioration of the column (affecting shape and retention time of peaks, mainly GPS). It was resolved using 24 mM of N-ethylmorpholine and 16.4 mM of glacial acetic acid instead.

In further studies Rombaut et al. [94] and Rodríguez-Alcalá and Fontecha [40] also used silica columns (3 and 5 *μ*m, resp.) attached to ELSD, adding formic acid (1 M) to the chloroform/methanol mobile phase used in the analysis of dairy products (cows', goats', and ewes' milk, cream, liquid and powder buttermilk, butter, whey, quark, and cheddar cheese) to increase the peak shape and resolution. Also, in the mobile phases a TEA buffer was used to adjust the final pH to 3 and avoid deterioration of the stationary phase. These authors did not report problems using these modifiers.

In the method by Rombaut et al. [94], neutral lipids are not eliminated in the samples but these moieties eluted separately enough as to enable the quantification of PA, GPI, GPE, GPS, GPC, and SM. In subsequent studies these authors modified the mobile gradient (87.5 : 12 : 0.5 v/v/v chloroform/methanol/TEA buffer to 28 : 60 : 12) using only a line A with dichloromethane (less toxic than chloroform) and a line B with methanol and TEA buffer (500 : 21, pH 4.5) [95]. Nevertheless, in the assayed conditions is observed a slight loss of resolution for the SM peak that elutes immediately after GPC while in the original method this compound was separated into three peaks. This method has been recently adapted to the detection of PL by CAD in whole and skim milk, cream, butter, buttermilk, butter oil, and butterserum [42]. During the assays, it was found that using TMA buffer at pH 4.5 led to coelution of PA and GPI while a pH 3.5 separated these two compounds and increased the resolution of GPC and SM. Although in the modifications carried out by Lin et al. the limits of detection (LOD) were not reported, when comparing the values from the original method [94] with those using the adaptation for the HPLC-CAD analysis, it can be seen that this latter detector resulted in a generally higher sensitivity than ELSD as already mentioned above (e.g., LOD for GPS, 2.06 ng using CAD versus 18 ng using ELSD; LOD for GPC, 9.50 ng versus 11 ng). However, other authors using similar columns but a gradient involving ammonium formate (3 g/L) and acetonitrile/methanol (100 : 3 v/v) to analyse human milk PL obtained LOD of 0.1 ng for GPI, 0.4 ng for GPE, 0.3 ng for GPS and GPC, and 0.5 ng for SM [96].

On the other hand, Rodríguez-Alcalá and Fontecha [40] combined the chloroform/methanol/TEA buffer gradient, previously assayed by Rombaut et al. [94], adding isooctane/THF 99 : 1 (v/v) and a regeneration step using isopropanol. Under these conditions it was possible to resolve neutral lipids into CE, TG, DG + CH + FFA, MG, and PL into GlucCer, LacCer, PA, GPE, GPI, GPC, and SM. In further research works aiming at the total milk fat extraction and quantification of polar and neutral lipids of cows', goats', and ewes' milk by ASE, the gradient of this latter HPLC method was modified while two silica columns instead of one were used, being able to separate DG from CH and FFA [37].

Nevertheless, the analysis of PL is not limited to classic approaches and it is evolving rapidly in the last years. A novel technique based on the derivatization of polar and neutral amino acids (AA) with 9-fluorenylmethyl chloroformate (FMOC) has been adapted to the determination of GPE in powder and liquid dairy products [97]. Thus, after treatment of samples with CM mixtures, the solvent is placed into a vial and the reaction is performed by the HPLC autosampler. For the elution of the compounds, a gradient of chloroform/methanol/25% ammonium hydroxide from 80/19.5/0.5 (v/v/v) to chloroform/methanol/water/25% ammonium hydroxide 60/34/5.5/0.5 is used while the detection is carried out by a fluorescence detector (FLD) at excitation wavelength of 265 nm and emission of 305 nm. In the assayed conditions LOD and LOQ for milk powder were estimated as 16 mg/kg and 53 mg/kg, respectively, while for commercial liquid milk as 15 mg/kg and 49 mg/kg. This derivatization reaction can also be applied to the quantification of GPS, LPE, and LPS.

Furthermore, hydrophilic interaction chromatography (HILIC) that combines hydrophilic stationary phases with reversed-phase type eluents has been applied for the determination of free choline, GPC, phosphocholine, phosphatidylcholine, LPC, and SM in 48 commercial dairy products, soy and almond beverages and artificial creamer by a QTRAP mass spectrometer operating at ion scan, neutral loss scan, and multiple reaction monitoring (MRM) [98].

HILIC chromatography is especially suitable for working with mass spectrometers: it provides providing larger diffusion constants of analytes during their migration through the column and also better ionization efficiency in electrospray ionization (ESI) as compounds elute in order to increase hydrophilicity but using exclusively organic solvents, thus avoiding extremely water-rich, or ion-pair containing mobile phases used under reversed-phase mode. ESI gives full information of the intact molecule while structural analysis can be obtained via MS/MS. On the basis of these concepts elsewhere the molecular species of PL identified in cows' and donkeys' milk (HILIC-HPLC MS-ESI-IT-TOF) has been reported after isolation with SPE and separation by a partially porous column (Fused-core, 2.7 *μ*m) while the quantification was carried out using ELSD [99]. In subsequent research works these authors improved the method to bypass the SPE step [100].

## 7. Concluding Remarks

Phospholipids are compounds of a high interest from both technological and biological points of view. Milk and dairy foods are natural rich sources of these compounds and valuable since PS and SM are only presented in animal products but some of them are restricted to assure the health of the consumer. In the analysis of PL, HPLC methods using ELSD, CAD, or MS detectors have been established as reliable and simple protocols that can bypass the cumbersome steps of fractionation and purification. In future research works, these protocols must be combined with others able to obtain information of the distribution and fatty acid composition of each PL in order to collect a profound comprehension and be able to identify those with the most interesting capabilities.

## Figures and Tables

**Table 1:** PL content in dairy products.

	mg/100 g product	g/100 fat	Reference
Ewes' raw milk	29.8	0.390	[33–38, 40, 41]
Goats' raw milk	27.6	0.710	
Cows' raw milk	29.4–40.0	0.700–0.980	

Skimmed milk	20.0–81.9	11.1–19.1	[42]

Pasteurized semiskimmed	18.8	1.30	[34]
UHT full fat	21.2	0.600	
UHT semiskimmed	14.2	0.900	
UHT skimmed	12.8	10.7	
Sterilized semiskimmed	16.0	1.00	

Infant formula (0–6 mo.)	228–304	NR	[43]
Infant formula (0–3 mo.)	607	NR	

Nonsweetened condensed milk	75.1	1.00	[34]
Cream	139–190	0.350–0.860	[33–35, 39]
Butter	70.5–230	0.090–0.270	[33–35, 42]
Butteroil	10.0	0.0100	[42]
Buttermilk	1.12–160	4.49–33.1	[33–35, 39, 44]
Buttermilk (skimmed)	110	20.00	[92]

Buttermilk (acid)	160	33.05	[33–35]
Buttermilk (reconstituted)	130	21.66	
Buttermilk quark	310	29.06	
Butterserum	660–1250	14.8–48.4	

Butterserum (whole)	97.0	40.0	[92]
Butterserum (skimmed)	93.0	49.0	

Goat buttermilk	NR	0.19	[39]
Goat butterserum	NR	1.010	

Fermented buttermilk-sweet	91.8	21.8	[33–35]
Fermented buttermilk-sour	115.5	23.1	
Yoghurt skimmed	17.9	5.50	
Kefir semiskimmed	34.0	2.30	
Whey (cheddar)	18.0	5.32	
Whey (emmental)	22.0	45.2	
Fresh cheese	310	29.1	
Ricotta	279	2.70	
Quark-skimmed	32.4	24.70	
Quark-cream	58.1	0.90	
Cottage cheese	55.8–376	1.30–5.30	
Sphingolipid quark mix	480	10.60	
Fresh cheese	149	0.600	
Mozzarella (buffalo)	115	0.500	
Gouda light 8 weeks	93.9	0.400	
Gouda 8 weeks	151	0.500	
Gouda 36 months	147	0.400	
Cheddar	154	0.500	
Camembert	123	0.500	
Emmental	110	0.400	
Parmigiano 24 months	111	0.400	
Mozzarella (whey)	19.1	6.20	
Cheddar (whey)	17.6	5.30	

**Table 2:** PL species distribution (g/100 g PL) in dairy products.

	GPE	GPC	GPS	GPI	SM	Reference
Ewes' milk	26.1–40.0	26.4–27.2	4.96–10.7	4.16–6.40	22.6–29.7	[36–38, 40, 41]
Goats' milk	19.9–41.4	27.2–31.9	3.2–14.0	4.00–9.37	16.1–29.2	
Cows' milk	23.4–46.7	19.1–33.2	2.00–9.07	3.98–8.97	17.8–29.2	

Pasteurized semiskimmed	35.0	20.2	8.90	7.90	17.0	[34]
UHT (full fat)	34.0	20.5	9.10	7.90	19.5	
UHT (semiskimmed)	33.0	22.0	7.90	4.80	22.9	
UHT (skimmed)	38.2	19.6	9.90	5.50	16.7	
Sterilized milk (semiskimmed)	34.3	24.2	7.70	5.10	17.4	

Buttermilk (powder)	19.8	33.9	20.6	4.93	19.9	[40]
Buttermilk	33.5–39.5	27.71–35.5	10.3–22.8	2.40–7.20	16.7–18.3	[39, 44]
Butterserum	27.2	29.8	7.20	10.8	24.9	[39]
Butteroil	20.3	50.7	ND	ND	25.9	[42]

Goats' buttermilk	35.2	24.8	9.90	9.80	20.3	[39]
Goats' butterserum	27.1	26.2	8.20	11.7	26.8	

Yoghurt (skimmed)	31.1	19.9	7.90	6.30	24.9	[34]
Fresh cheese	39.9	21.7	8.20	6.50	14.1	
Cheddar	38.0	20.3	8.50	7.70	16.3	
Cheese whey (cheddar, mozzarella)	40.6–41.1	19.0	9.30	3.70–4.60	15.7–16.4	

Cream	42.7	14.6	7.20	6.80	28.6	[39]
Butter	31.0	24.7	15.3	11.9	17.1	

Infant formula (0–6 mo.)	23.2–29.5	25.9–35.6	5.51–10.1	10.2–19.5	13.1–26.9	[43]
Infant formula (0–3 mo.)	23.6	41.7	4.28	9.88	20.6	

**Table 3:** Palmitic, stearic, and oleic content (g/100 g FA) in PL from dairy products.

	Palmitic acid	Stearic acid	Oleic acid
Ewes' milk			

GPC	34.8	18.9	32.3
GPS	28.8	33.9	16.7
GPI	25.5	28.6	25.2
GPE	12.0	17.5	53.6
SM	25.0	6.30	0.60

Goats' milk			

GPC	40.0	8.00	34.4
GPS + GPI	13.3	47.9	29.7
GPE	21.2	12.4	50.6
SM	20.7	8.00	0.90

Cows' milk			

GPC	34.7	9.5	26.7
GPS + GPI	27.7	25.7	24.9
GPE	35.5	16.9	23.5
SM	18.9	4.30	0.50

Data from Zancada et al. [38].
